# The Role of COVID-19 in Excess Mortality in Slovakia: A Novel Approach Based on Healthcare Billing Records

**DOI:** 10.3389/ijph.2024.1607537

**Published:** 2024-12-03

**Authors:** Peter Barančok, Ján Dudek, Petra Ištokovičová, Daniela Kandilaki, Michal Kotrč, Ľubica Löffler, Matej Mišík, Roman Mužik, Beáta Saal, Martina Vrbiková, Rudolf Zajac, Martin Selvek, Peter Pažitný

**Affiliations:** ^1^ Institute for Healthcare Analyses, Ministry of Health, Bratislava, Slovakia; ^2^ White Plume Technologies, Birmingham, AL, United States; ^3^ Faculty of Management, Prague University of Economics and Business, Prague, Czechia; ^4^ Union Health Insurance Company, Bratislava, Slovakia; ^5^ Independent Healthcare Consultant, Bratislava, Slovakia; ^6^ Dôvera Health Insurance Company, Bratislava, Slovakia; ^7^ Reporting and Health Statistics, National Health Information Center, Bratislava, Slovakia

**Keywords:** COVID-19, Slovakia, excess mortality, COVID-19 confirmed death, COVID-19 associated death

## Abstract

**Objectives:**

Propose a methodology to identify COVID-19 associated deaths using healthcare billing records and evaluate its effectiveness by comparing the results with excess mortality data from 2020 to 2022 and confirmed COVID-19 deaths.

**Methods:**

A retrospective quantitative analysis was conducted by merging healthcare billing records with cause of death data. The term “COVID-19 associated death” was defined as any death occurring within a defined timeframe following a confirmed contact with COVID-19. This category includes individuals who died directly due to COVID-19, with COVID-19 as a contributing factor, or as an aftermath of a COVID-19 infection, as well as those who died from other causes but had previously contracted COVID-19. This broader definition provides a more comprehensive measure of excess mortality compared to the officially confirmed COVID-19 deaths attributed to the virus.

**Results:**

We identified 35,399 COVID-19 associated deaths during the 3-year pandemic in Slovakia compared to 21,395 confirmed COVID-19 deaths.

**Conclusion:**

The identification of COVID-19 associated deaths with our methodology offers a more accurate explanation for the notably high excess mortality observed in Slovakia (31,789 deaths) during the pandemic, relative to the EU27. Given the high level of excess mortality, the officially confirmed deaths are likely underestimated, and the presented methodology provides a more precise measure of mortality. Additionally, healthcare billing records prove valuable in identifying these deaths at the individual patient level using claims data of health insurance companies, which is crucial for implementing targeted preventive measures and improving preparedness for future pandemics.

## Introduction

The COVID-19 pandemic affected EU member states differently, with some countries experiencing more severe outbreaks than others. Due to diverse protocols employed by countries for testing and documenting COVID-19 deaths, comparing data across countries is complex. As a result, numerous institutions have opted to use alternative indicators, such as excess mortality or loss of life expectancy, to more accurately measure the pandemic’s impact [1–3].

We use the 2016–2019 monthly average mortality in Slovakia as the baseline for our analysis. Eurostat has published data on excess mortality, expressed as a percentage of additional deaths each month compared to a monthly average from the period 2016–2019. Slovakia maintained a stable death rate between 2011 and 2019 [4], making the 2016–2019 average a suitable baseline for calculating excess mortality.

Compared to the EU-27 average, Slovakia experienced significantly higher excess mortality, reaching 19.2% versus the EU-27 average of 12.3% for the period 2020–2022 [5]. In absolute terms, this equates to 31,789 excess deaths over 3 years compared to the baseline period of 2016–2019. Slovakia also saw one of the most significant declines in life expectancy at birth in 2021 compared to 2019 (Table 1).

**TABLE 1:** Deaths and excess mortality in Slovakia and EU-27.

	2016–2019 annual average	2020	2021	2022	2020–2022 total
Deaths Slovakia (people with permanent residence in Slovakia)^a^	53,448	59,089	73,461	59,583	192,133
Excess mortality Slovakia^a^	-	5,641	20,013	6,135	31,789
Confirmed COVID-19 deaths^a^	-	4,004	14,769	2,723	21,496
Excess mortality indicator (%) Slovakia^b^	-	10.4%	35.7%	11.4%	19.2%
Excess mortality indicator (%) EU 27 average^b^	-	11.7%	14.0%	11.1%	12.3%
Change in life expectancy at birth compared to 2019 (years) Slovakia^c^		−0.80	−3.20	−0.80	
Change in life expectancy at birth compared to 2019 (years) EU 27^c^		−0.90	−1.20	−0.70	

^a^ Statistical Office SR, 2024 (Data extracted on 06/02/2024 from DATAcube.

^b^ Eurostat, Excess mortality indicator 2024 (extracted on 06/02/2024 from ESTAT.

^c^ Eurostat, Life expectancy by age and sex (extracted on 04/08/2024 from ESTAT.

During the first wave of the COVID-19 pandemic in the spring of 2020, Slovakia managed to avoid the severe outcomes seen in countries like Italy and recorded very low mortality rates [6]. However, the second wave in the fall of 2020 severely impacted Slovakia, with excess mortality rising sharply, above the EU-27 average. While mortality returned to lower levels in the summer of 2021, it spiked in the autumn of 2021, with excess mortality reaching 35.7% (compared to 14.0% EU average) alongside a significant decline in life expectancy at birth. In 2022, Slovakia’s excess mortality was close to the EU-27 average, but remained 11.4% higher than the baseline period of 2016–2019.

While data on excess deaths suggests that COVID-19 was associated with a significant increase in mortality, this measure has several limitations. Most importantly, excess deaths express a deviation in the number of deaths from a baseline but do not directly attribute any specific cause to those deaths. While COVID-19 undoubtedly directly caused many of these excess deaths, other factors may have contributed, such as reduced access to emergency care or fewer preventive care visits [7]. Conversely, deaths for some causes, such as car accidents, may have decreased due to reduced commuting or travel during lockdown periods [8, 9].

As described in greater detail in the Methods section, our methodology utilizes data from healthcare billing records and official causes of death to differentiate between deaths unrelated to COVID-19 and those associated with it. The aim is to better estimate COVID-19 related mortality in Slovakia, and we believe this method could be applied to calculate the death toll in other countries as well.

As discussed earlier, excess mortality has certain drawbacks, namely that it provides a population-wide perspective without the ability to specifically identify which patient groups fall into the excess mortality category and which individuals would have died regardless. On the other hand, healthcare billing records with ICD (International Classification of Diseases) codes can be used to identify chronic diseases in individual patients and specific disease populations [10]. Therefore, we sought a more effective method to identify COVID-19 related deaths using healthcare billing records to investigate the role of COVID-19’s in the 31,789 excess deaths in Slovakia from 2020 to 2022 compared to the baseline period.

## Methods

Our methodology is based on a retrospective analysis that utilizes healthcare billing records in combination with a dataset of death causes for the three full years from 2020 to 2022. We introduce the term “COVID-19 associated deaths” to describe deaths occurring within a defined timeframe after confirmed contact with COVID-19. This category includes individuals who died due to COVID-19, with COVID-19 as a contributing factor, or as an aftermath of a COVID-19 infection, as well as those who died from other causes but had contact with COVID-19 prior to death. This definition extends beyond the term COVID-19 confirmed death, which specifically refers to deaths officially attributed to COVID-19 as the cause of death. In parallel, the number of COVID-19 associated deaths provides a means to further investigate the true impact of COVID-19 on mortality. For statistical analysis, we used the 4.1.0 version of “R” software [11].

### Healthcare Billing Records

The dataset of healthcare billing records was sourced from health insurance companies (HICs), which maintain payment records for individual healthcare providers for services provided to the insured population. According to a methodological manual [12], HICs report [13] these billing records to the National Health Information Center (NHIC) on a monthly basis.

The Institute for Healthcare Analyses (IHA) of the Ministry of Health of the Slovak Republic, serving as an external collaborator of NHIC was granted access to this dataset, which includes all billing records for the entire Slovak population from 2020 to 2022.

### Dataset of Death Causes

The dataset of death causes contains information about all deaths collected by the Statistical Office of the Slovak Republic (SO SR), which is shared with the NHIC and accessed by IHA. The dataset includes details such as the deceased’s age, date of death, and reported cause of death, all of which is originally sourced from the deceased’s examination letter and the statistical death report. The reporting units are registry offices, which must verify the reliability and completeness of the data provided by the doctor during the examination or autopsy of the deceased, before submitting the statistical report to the SO SR [14].

When processing this dataset, two steps where undertaken. First, deaths of individuals who were not Slovak citizens (635 in total) where excluded. Second, duplicate person identifiers (2 in total) where excluded. After these data cleaning operations, 192,131 deaths remained subject for analysis (see Supplementary Material S1).

### COVID-19 Associated Death

We define a COVID-19 associated death as the death (D) of an individual diagnosed with a COVID-19, occurring within a defined timeframe (T) after confirmed contact (C) with COVID-19.

#### D (Death)

A total of 192,133 deaths were recorded in Slovakia between 2020 and 2022, of which 190,557 were successfully linked to corresponding healthcare billing records (Supplementary Material S2). 1,576 deaths (0.8% of all deaths) could not be linked, likely representing Slovak citizens who were not insured within the public health insurance system in Slovakia.

The Slovak edition of the International Classification of Diseases (MKCH-10-SK) includes four codes for reporting COVID-19 cases [15]. Codes U07.3 and U07.4 were introduced in Slovakia for billing and reporting purposes following widespread antigen test introduction [16]:

• U07.1: used for patients with COVID-19 confirmed by laboratory testing. The code is applied whenever COVID-19 has been confirmed through laboratory tests, regardless of the severity of clinical symptoms or manifestations.
• U07.2: used for patients with a suspected COVID-19 diagnosis. We excluded this code from our analysis because it was used only when there was a suspicion of COVID-19 or when the virus was diagnosed without a clear laboratory test result [17].
• U07.3: used for COVID-19 infections confirmed by a PCR test.
• U07.4: used for COVID-19 infections confirmed by a certified antigen test.

For hospitalized patients, both the diagnosis at admission and at discharge were considered. If either diagnosis indicated COVID-19, the hospitalization was recorded as a contact with COVID-19. Of the 21,496 reported COVID-19 deaths, we were able to link 21,475 to healthcare billing records (Supplementary Material S2).

#### C (Contact)

Each contact was defined as healthcare services provided to a patient with a U07.1, U07.3, and U07.4 diagnosis for confirmed COVID-19 infection that was reimbursed by public health insurance companies.

For inpatient care, the date of admission was considered the date of contact, for other types of care it was the date when the care was provided.

In total, there were 3,162,879 confirmed COVID-19 contacts from 2020 to 2022. Notably, some patients were reported with double diagnosis (e.g., both U07.1 and U07.3, or U07.1 and U07.4 simultaneously) (Supplementary Material S3). It is also important to note that a group of individuals paid for their testing out-of-pocket. These self-payers were not included in the data, as health insurance companies do not maintain records about out-of-pocket self-paid healthcare services.

#### T (Timeframe)

Our methodology links deaths (D) to COVID-19 contacts (C) if they occur within a defined timeframe following the COVID-19 contact. We define these deaths as COVID-19 associated deaths to distinguish them from officially confirmed COVID-19 deaths. Given the significant differences in COVID-19 variants [18], their case-fatality ratio [19], and the availability of vaccines and treatments during the pandemic [20], it is important to calculate this timeframe separately for each wave.

The timeframe was tested for the three major COVID-19 waves. Interval I covers the period from 01/01/2020 to 27/06/2021, covering the period when the alpha variant was dominant as well as earlier periods when there were relatively few cases at the start of the pandemic in Slovakia; interval II covers the period from 28/06/2021 to 16/01/2022, during which the delta variant was dominant; and interval III covers the period from 17/01/2022 to 31/12/2022, when the omicron variant was dominant [21].

**FIGURE 1:**
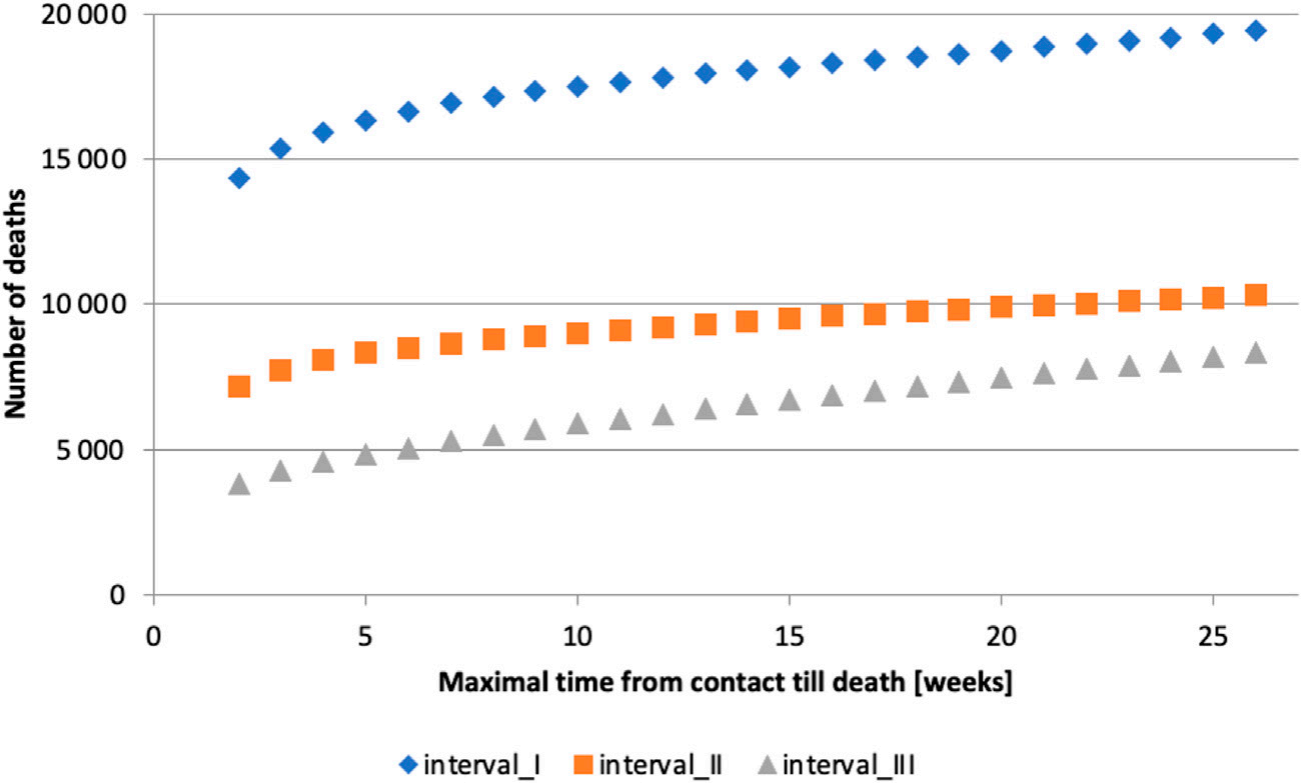
Maximum time from contact to death [weeks] (calculation of authors, Slovakia, 2024).

**FIGURE 2:**
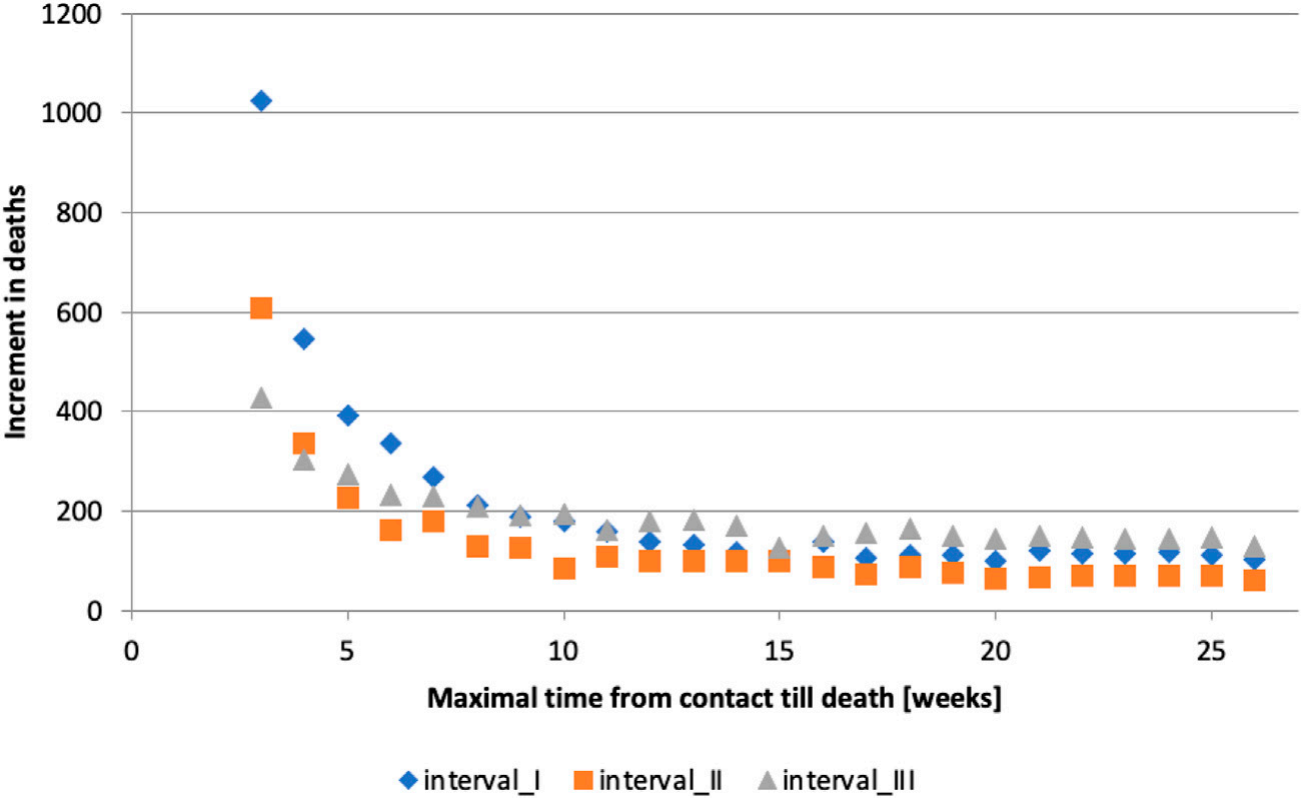
Maximum time from contact to death [weeks] (calculation of authors, Slovakia, 2024).

If the timeframe is too short, many deaths caused by or linked to COVID-19 will be omitted. Conversely, if the timeframe is too long, deaths unrelated to COVID-19 infection will be included. It can be observed that the number of deaths grows roughly linearly for long timeframes (Figure 1), which we hypothesize is due to deaths unrelated to COVID-19 infection. This observation guided us in determining the appropriate length of time between contact (C) with the healthcare system due to confirmed COVID-19 infection and subsequent death (D). The analysis was conducted in the following steps:

1. All contacts that occurred in interval I with a subsequent death were selected, and time until death was calculated.
2. The number of deaths that occurred within 14, 21, …, up to 182 days after the contact $d_{14},d_{21},...,d_{182}$ , was computed. (Note: The timeframe was restricted to multiples of 7 days - 1 week - to minimize the bias introduced by weekends.)
3. The increase in the number of deaths was calculated by extending the timeframe by 1 week increments (Figure 2): $\delta_{t}=d_{t}-d_{t-7},for\;t=21,28,...,182$

4. A simple linear regression model of the form $\delta =\alpha_{0}+\alpha_{1}\cdot t$ was fitted to the increments in deaths, $\delta_{21},\delta_{28},...,\delta_{128}$ , computed in step 3 and the significance of the coefficient $\alpha_{1}$ was determined.
5. Based on whether the coefficient $\alpha_{1}$ was significant at the level of 0.1% (P-value of 0.001 – Supplementary Material S4), two options where considered:
a. If the coefficient was significant, step 4 was repeated excluding the increment corresponding to the smallest t (starting with $\delta_{14}$ , then $\delta_{21}$ and so on).
b. If the coefficient was not significant for a given timeframe $\hat{t}$ , then the resulting timeframe is $T=\hat{t}-7$ and the analysis is completed.

Using the timeframe allows for assignment of each death as either COVID-19 associated death (occurring within timeframe) or non-associated death (occurring outside timeframe). The choice of p-value at 0.001 (a standard threshold used for stricter testing of statistical significance) aims to cover most of the COVID-19 associated deaths (high sensitivity) at the cost of also including more of the deaths randomly occurring during the timeframe (lower specificity).

Under the assumption that deaths not associated with COVID-19 (“random”) linearly increase with the length of the timeframe (which is not possible to validate for short timeframes from the available data), an estimate of the overall number of COVID-19 associated deaths that takes into account the presence of these “random” deaths could be produced. The asymptote of the “random” deaths could be fitted and subtracted from the deaths occurring after COVID-19 contact. While producing a populational estimate, this approach does not allow for assignment of each death as either COVID-19 associated death or non-associated death.

This analysis was initially performed for contacts from interval I, followed by contacts from interval II, excluding deaths associated with contacts from interval I. Finally, it was conducted for contacts from interval III, excluding deaths associated with contacts from intervals I and II.

As our dataset only included deaths up to the end of 2022, not all contacts from interval III could be used to compute of the length of the timeframe. This limitation arose, because for some contacts, the full length of the timeframe would not have been observable for longer timeframes (e.g., for contacts in the first week of December 2022, only a few weeks are observable), potentially causing an artificial decrease in deaths associated with COVID-19. Therefore, only contacts for which the longest considered timeframe of 182 days was fully observable, i.e., those that occurred up to 02/07/2022, were considered when determining the length of the timeframe. Once the appropriate timeframe length was determined, all contacts were included to associate deaths with COVID-19.

The length of the timeframe was determined to be 70 days for interval I, 98 days for interval II, and 63 days for interval III (Supplementary Material S5). Not surprisingly, the timeframe for interval III (Omicron) is shorter than for interval II (Delta), as the Omicron variant had a less severe clinical course and lower risk of death compared to the Delta variant [22].

In the analysis described above, we did not distinguish between genders or age groups of those infected. To justify the assumption, that the time from contact to death is not affected by age and gender, we examined the associations between these factors for the deaths identified by our method. Our rationale is that if age or gender had an observable effect, such effects would also be evident within the deaths identified by our method.

Since each COVID-19 associated death identified by our method could be preceded by multiple contacts, resulting in multiple potential times from contact to death, we chose the longest time that was still within the defined timeframe for the respective interval for the analysis. This was used as an approximation of the duration between the start of the infection and the subsequent death, since healthcare billing records do not contain information on the exact time of infection, and accurately estimating this from the records is both difficult and impractical.

To determine whether there is an association between age and time to death we calculated Pearson correlation coefficients for each interval (Supplementary Material S6).

A very weak negative correlation was observed.

To determine if there is an association between sex and time to death we performed a Kolmogorov-Smirnov test to compare the distributions of times for women and men (Supplementary Material S7).

It can be observed that the distributions do not appear to be significantly different at the 0.001 level of significance, which was used for determining the timeframes. (Note: The difference between the distributions for interval I would be significant at the level of 0.05, although only nearly. However, we consider this level to be too lenient and chose to maintain consistency with the stricter confidence level.)

Based on the Pearson correlation coefficient and the results of the Kolmogorov-Smirnov test, we do not find it necessary to differentiate between genders or age groups of the infected when determining the timeframe.

### Limitations

There are several limitations to our analysis.

First, our methodology counts deaths that occurred within the specified timeframe after contact with a diagnosis confirming a COVID-19 infection but does not verify whether the infection directly or indirectly contributed to the death.

Second, it does not include deaths that occur after the defined period following contact. Since we use specific timeframes (70 days in interval I, 98 days in interval II, 63 days in interval III) within which a death from contact with COVID-19 must occur, our methodology naturally excludes deaths occurring after these timeframes, even if caused by COVID-19.

Third, the information on COVID-19 tests in our databases is incomplete. Tests paid for by self-payers are not included (as HICs do not reimburse these tests), and the results of these tests are unknown. This could result in missing data on patients who were COVID-19 positive, although such patients could still be assigned a COVID-19 diagnosis in billing records for care provided by means other than testing. Additionally, healthcare services provided to individuals outside the public health insurance system are not reported and, therefore, not accounted for in our analysis.

Finally, the dataset of healthcare billing records does not include information on secondary diagnoses during patient hospitalizations. As a result, hospitalized patients admitted with a different diagnosis, where COVID-19 could have been a contributing factor, may not be included in our count of subsequent contacts and deaths (deaths with COVID-19 or its aftermath) if they did not have recorded contact with COVID-19 in other care settings.

## Results

The total number of COVID-19 associated deaths was 35,399. This figure includes 17,495 deaths with contact from interval I, 9,410 from interval II and 8,492 from interval III (Supplementary Material S8).

**FIGURE 3:**
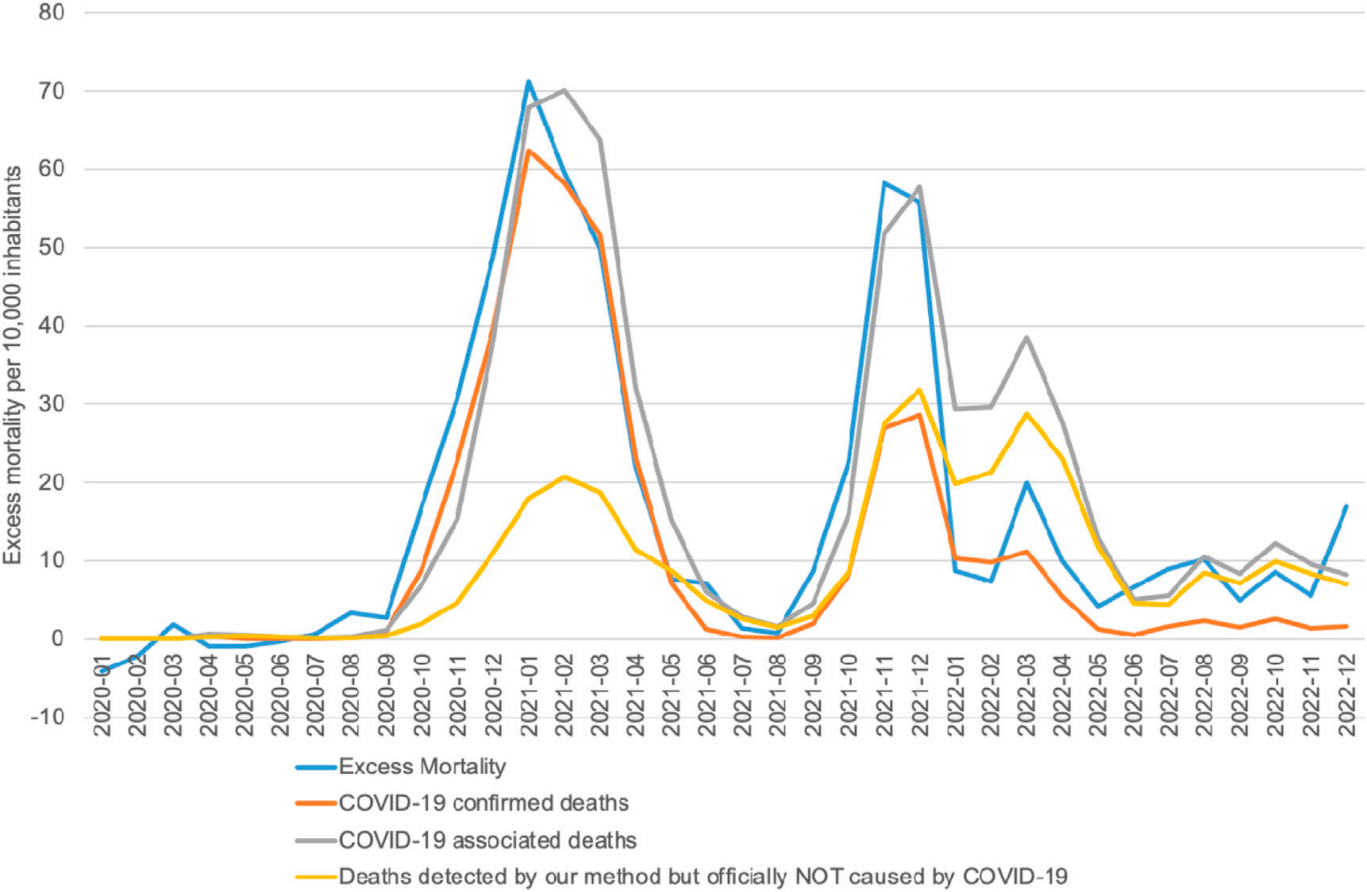
COVID-19 associated deaths vs COVID-19 confirmed deaths vs Excess mortality (calculation of authors, Slovakia, 2024).

Identifying COVID-19 associated deaths provides a closer approximation to the notably high excess mortality observed in Slovakia during the pandemic, compared to the EU-27. Our methodology identified 35,399 COVID-19 associated deaths in contrast to 21,395 confirmed COVID-19 deaths, and aligns more closely with the observed excess mortality (31,789 deaths), as shown in Figure 3. Our findings suggest that the impact of COVID-19 on excess mortality was much greater than indicated by the official COVID-19 death statistics, particularly evident during the second peak, where the number of confirmed COVID-19 deaths explains only half of the overall excess mortality.

**FIGURE 4:**
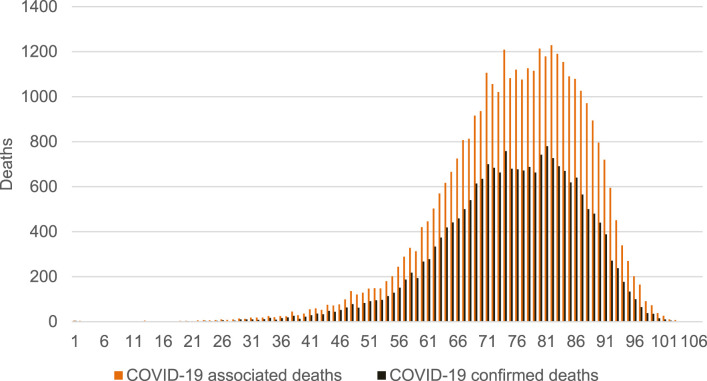
Age structure of COVID-19 associated deaths vs COVID-19 confirmed deaths (calculation of authors, Slovakia, 2024).

COVID-19 was particularly deadly for older populations (Figure 4). In terms of age structure, our approach identified 28,926 deaths among individuals older than 65 years, representing 81.7% of all COVID-19 associated deaths. This is compared to 80.7% of people 65 and above among COVID-19 confirmed deaths, and a 77% share of people aged 65+ in the total number of deaths (Supplementary Material S9).

The average age of men at the time of death (Supplementary Material S10) was lower compared to women (Supplementary Material S11), reflecting the situation in Slovakia, where women tend to live to a higher average age [23]. The distributions for the three groups of COVID-19 confirmed deaths, COVID-19 associated deaths, and COVID-19 associated deaths that are not among the COVID-19 confirmed deaths were approximately the same.

## Discussion

Our approach identified 35,399 COVID-19 associated deaths compared to 21,395 COVID-19 confirmed deaths during 2020–2022 (Supplementary Material S12). When considered in the context of 31,789 excessive deaths, it suggests that COVID-19 had a much greater impact on the excess mortality then previously presumed, based on existing reports and research.

In this regard, we highlight two groups of deaths (Table 2):

1. Unrecognized COVID-19 associated deaths: A group of 18,017 individuals who had contact with a COVID-19 infection prior to their death, but whose official cause of death was not listed as COVID-19.
2. COVID-19 confirmed deaths without documented contact: A group of 4,013 people, who were officially recorded as having died due to COVID-19, but for whom there was no documented COVID-19 contact in their healthcare billing records. Further analysis revealed, that within this group, 2,684 were only suspected of having COVID-19 (Dg. U07.2), 1,260 had no COVID-19 diagnosis in their healthcare billing records, and 69 were excluded due to timeframe limits because the time between the contact and death was longer that the defined timeframe in the given interval (Supplementary Material S13). The identification of only 69 such cases underscore the strength of our methodological approach.

**TABLE 2:** COVID-19 associated deaths and COVID-19 confirmed deaths (calculation of authors, Slovakia, 2024).

	COVID-19 confirmed deaths	Other causes of death	Total
COVID-19 associated deaths (contact with COVID-19 before death, dg. U07.1, U07.3, U07.4)	17,382	18,017	35,399
No contact with COVID-19 or U07.2 (suspicion on COVID-19)	4,013	152,719	156,732
Total	21,395	172,130	192,131

The previously unrecognized 18,017 deaths represent a substantial portion (51%) of all COVID-19 associated deaths, nearly matching the number of COVID-19 confirmed deaths (21,395). Further analysis of these deaths could provide valuable insight into specific groups that may need better protection during a pandemic.

As these individuals had a COVID-19 diagnosis in their healthcare billing record but not as their cause of death, it is possible that their pre-existing health conditions worsened due to COVID-19, leading to their death. COVID-19 has been shown to increase mortality rates, particularly among patients with highly prevalent diseases, such as cardiovascular disease, diabetes mellitus, morbid obesity and cancer [24–26]. High-risk patients, especially those with multiple clinical conditions face significantly higher-than-average risks even after receiving COVID-19 vaccinations. A study conducted in the United Kingdom found that the likelihood of severe outcomes, including death was about 4.82 higher for people with five or more clinical conditions than for those with one underlying clinical condition [27]. The use of healthcare billing records allows for identification of these pre-existing health conditions in COVID-19 associated deaths, and thus to identify the populations in higher risk of COVID-19 related deterioration of health and death.

Furthermore, in this group, a total of 15,426 deaths were among individuals aged 65 or older, representing almost 86% of these unrecognized COVID-19 associated deaths. This is comparable to data from the United States in 2020, where 82% of unrecognized deaths were among persons aged 65 and older [28].

The ability to identify these deaths, at least partially, from billing records databases makes them valuable for future pandemic response planning. Improving the real-time availability of these databases as well as digitalization of death certificates could enhance future pandemic responses [29]. This is particularly important in Slovakia and other CEE countries with low-performing healthcare systems and higher case-fatality ratios from COVID-19 compared to countries with more advanced healthcare systems. Countries that struggle to provide high-quality care of chronic patients are unlikely to achieve better outcomes with COVID-19 patients [30].

Several studies have utilized Korean health insurance data to study COVID-19 deaths, but they primarily focused on the impact of comorbidities on the probability of death from COVID-19 rather than on the actual number of COVID-19 deaths [31, 32]. Combining their findings with a more accurate identification of COVID-19 associated deaths could yield more valuable results. The authors are not aware of another study that has developed a new method to count COVID-19 associated deaths.

Most studies estimating the number of COVID-19 deaths have relied on data from national statistical offices to compare the officially reported COVID-19 deaths to the total excess mortality and almost all find official COVID-19 death counts to be underestimated [33–35].

Supplementary Material S14 shows that there were 122,300 excess deaths in the US between March and May 2020 when compared to previous years [34]. However, only 95,235 deaths were reported as COVID-19 deaths, leading to the authors to conclude that COVID-19 deaths were underestimated by 28.4% [34]. Others found that for all of 2020, the US COVID-19 death count was potentially underestimated by 38.2%. In Slovakia comparable figures were observed for 2020, with a potential underestimation of COVID-19 deaths of 40.9%. This number decreased to 35.5% in 2021 but significantly increased to 125.3% for 2022. However, the course of the illness in 2022 was significantly different due to vaccines, anti-viral drugs, and other factors, which may have reduced the lethality of the disease.

An analysis of Italian excess mortality revealed an even starker underestimation of COVID-19 deaths by as much as 60%. Official reporting in the country was incomplete, for example, completely missing fatalities from nursing homes [35].

The new methodology proposed in this paper can help mitigate the significant issue of COVID-19 death underestimation by focusing on patient contact with COVID-19 rather than relying solely on excess mortality data. Additionally, identifying COVID-19 associated deaths allow for a more granular, individual-level analysis, not just a population-wide perspective. When combined with further information obtained from billing records, this approach can be a valuable tool for directed and data-driven decision making during future health emergencies. Early identification can lead to better-targeted preventive measures and improved case management. Finally, healthcare billing data has proven to be a reliable, precise and timely source of information, suggesting that methodologies based on these data may become crucial for implementing targeted preventive measures and improving preparedness for future pandemics.
