# Supplementary material for: The Role of COVID-19 in Excess Mortality in Slovakia: A Novel Approach Based on Healthcare Billing Records
Source: Int J Public Health. 2024 Dec 3;69:1607537. doi: 10.3389/ijph.2024.1607537 (PMC11649408; doi:10.3389/ijph.2024.1607537)
Supplement: Supplementary file 1 [file DataSheet1.pdf]

Supplementary File 1: Deaths total **(calculation of authors, Slovakia, 2024)**

**Year**	**Slovak residency**		**Residency not in Slovakia**		Total	
	**Deaths total**	**Out of which dg U07**	**Deaths total**	**Out of which dg U07**	**Deaths total**	**Out of which dg U07**
2020	59,089	4,004	174	3	59,263	4,007
2021	73,461	14,769	192	19	73,653	14,788
2022	59,583	2,723	269	6	59,852	2,729
	192,133*	21,496	635	28	192,768	21,524

* Note: Out of these 192,133 deaths, there were 2 mistakes in ID

Supplementary File 2: Causes of death, all persons, and persons with health insurance **(calculation of authors, Slovakia, 2024)**

Month	All persons	Persons with health insurance					
		All causes	U07.1	U07.2	U07.3	U07.4	All codes for reporting COVID-19
2020-01	4991	4891					0
2020-02	4690	4650					0
2020-03	4995	4954	1				1
2020-04	4282	4238	25				25
2020-05	4229	4179	5				5
2020-06	4057	4005	1				1
2020-07	4276	4228	1				1
2020-08	4436	4389	10				10
2020-09	4327	4292	57				57
2020-10	5389	5346	488				488
2020-11	6051	5995	1248				1248
2020-12	7366	7320	2159				2159
2021-01	9081	9032	3298	21	25	75	3419
2021-02	8040	7998	3043	17	63	62	3185
2021-03	7593	7540	2568	13	162	73	2816
2021-04	5512	5478	1031	3	180	45	1259
2021-05	4682	4642	296	4	71	19	390
2021-06	4450	4422	39		27	5	71
2021-07	4298	4264	12		4		16
2021-08	4280	4247	5		3	1	9
2021-09	4632	4599	56	1	40	10	107
2021-10	5673	5650	260	2	155	34	451
2021-11	7517	7492	853	11	482	126	1472
2021-12	7701	7668	930	7	489	138	1564
2022-01	5662	5611	311	6	206	44	567
2022-02	5192	5164	293	5	185	54	537
2022-03	5954	5906	324	6	219	62	611
2022-04	4851	4807	167		95	30	292
2022-05	4488	4425	39	1	26	6	72
2022-06	4416	4371	13		13	5	31
2022-07	4702	4660	38	1	28	20	87
2022-08	4781	4735	70		43	14	127
2022-09	4423	4386	34	1	36	14	85
2022-10	4897	4859	67		52	24	143
2022-11	4639	4591	42	2	26	8	78
2022-12	5578	5523	36		35	20	91
Total	192131	190557	17820	101	2665	889	21475

Supplementary File 3: Number of patients' contact with COVID-19 **(calculation of authors, Slovakia, 2024)**

**Year-Month**	**U07.1**	**U07.3**	**U07.4**	**confirmed COVID-19**	**U07.2**
2020-01					2
2020-02	7			7	3
2020-03	993			993	50 314
2020-04	1 203		1	1 204	46 312
2020-05	630			630	34 287
2020-06	203		1	204	20 074
2020-07	618	2		620	21 352
2020-08	2 063	12	1	2 076	30 436
2020-09	4 800	19	5	4 821	76 760
2020-10	35 856	51	10	35 904	228 622
2020-11	41 640	20	25	41 680	129 027
2020-12	104 706	14	42	104 746	297 419
2021-01	196 558	107	109	196 672	306 382
2021-02	187 461	1 555	2 378	189 625	297 003
2021-03	170 536	11 921	18 147	190 531	301 112
2021-04	72 192	7 841	11 421	87 219	179 232
2021-05	31 478	3 439	4 797	38 031	129 736
2021-06	14 063	1 099	1 412	16 111	87 550
2021-07	7 219	380	429	7 915	119 891
2021-08	7 055	611	508	7 934	115 374
2021-09	18 863	4 158	2 967	24 334	175 479
2021-10	53 532	17 206	11 464	75 813	316 482
2021-11	163 370	63 582	44 064	245 146	573 779
2021-12	128 728	59 301	26 265	191 019	375 079
2022-01	129 439	55 802	36 781	201 920	416 766
2022-02	312 836	132 551	115 877	507 541	670 858
2022-03	223 031	77 925	96 753	362 481	419 860
2022-04	72 760	23 153	30 669	117 065	185 424
2022-05	22 049	4 120	10 671	35 018	52 824
2022-06	16 833	2 278	9 470	27 155	29 035
2022-07	49 065	4 672	35 083	83 632	47 183
2022-08	52 727	5 003	42 895	94 908	47 334
2022-09	51 868	2 995	46 405	97 155	45 077
2022-10	51 641	2 778	45 851	96 134	48 061
2022-11	22 016	1 286	18 592	40 357	34 397
2022-12	19 761	1 153	16 809	36 278	32 149
total	2 267 800	485 034	629 902	3 162 879	5 940 675

Supplementary File 4: P-value **(calculation of authors, Slovakia, 2024)**

dt	interval_I	interval_II	interval_III
14	0,0003064	0,0002816	0,0000063
21	0,0003064	0,0002816	0,0000063
28	0,0000240	0,0000140	0,0000004
35	0,0000159	0,0000010	0,0000008
42	0,0000327	0,0000005	0,0000013
49	0,0000324	0,0000035	0,0000081
56	0,0000371	0,0000004	0,0000379
63	0,0001600	0,0000029	0,0002431
70	0,0008670	0,0000161	0,0013566
77	0,0038725	0,0000011	0,0076328
84	0,0203613	0,0000101	0,0091427
91	0,0866924	0,0000632	0,0368695
98	0,2640681	0,0004060	0,1709020
105	0,3334893	0,0026483	0,5440465
112	0,2930787	0,0141310	0,0154710
119	0,7402902	0,0723306	0,0098507
126	0,9140413	0,0565122	0,0190316
133	0,9054591	0,2887905	0,1020052
140	0,8256100	0,9023653	0,1995181
147	0,0321592	0,4803483	0,1076411
154	0,0972121	0,2221046	0,2745995
161	0,1114325	0,2864939	0,4079819
168	0,0244914	0,2809444	0,4613057

Supplementary File 5: Total deaths and increment of total deaths **(calculation of authors, Slovakia, 2024)**

	**Total deaths**			**Increment of total deaths**		
**dt**	**interval_I**	**interval_II**	**interval_III**	**interval_I**	**interval_II**	**interval_III**
14	14348	7147	3822			
21	15374	7756	4250	1026	609	428
28	15920	8093	4554	546	337	304
35	16311	8319	4829	391	226	275
42	16648	8482	5062	337	163	233
49	16917	8662	5291	269	180	229
56	17128	8790	5499	211	128	208
63	17316	8915	**5691***	188	125	192
70	**17495***	9000	5884	179	85	193
77	17655	9109	6045	160	109	161
84	17794	9210	6224	139	101	179
91	17926	9310	6407	132	100	183
98	18044	**9410***	6578	118	100	171
105	18157	9511	6703	113	101	125
112	18296	9600	6853	139	89	150
119	18401	9672	7008	105	72	155
126	18512	9759	7173	111	87	165
133	18622	9836	7323	110	77	150
140	18722	9900	7466	100	64	143
147	18841	9966	7617	119	66	151
154	18956	10036	7763	115	70	146
161	19071	10105	7906	115	69	143
168	19188	10176	8050	117	71	144
175	19298	10246	8198	110	70	148
182	19400	10306	8327	102	60	129

Note: * $p<0.001$

Supplementary File 6: Association between age and time to death [by interval] **(calculation of authors, Slovakia, 2024)**

Interval	Pearson correlation coefficient
Interval I	-0.04957
Interval II	-0.06546
Interval III	-0.07476

Supplementary File 7: Association between sex and time to death [by interval] **(calculation of authors, Slovakia, 2024)**

	P-value of K-S test (women vs. men)
Interval I	0.04790
Interval II	0.29560
Interval III	0.43424

Supplementary File 8: Number of COVID-19 associated deaths according to interval of contact **(calculation of authors, Slovakia, 2024)**

Year-Month	Interval I (dominantly alpha)	Interval II (dominantly delta)	Interval III (dominantly omicron)	COVID-19 associated Deaths	COVID-19 confirmed Death
2020-01					
2020-02					
2020-03	5			5	1
2020-04	33			33	25
2020-05	30			30	5
2020-06	15			15	1
2020-07	7			7	1
2020-08	14			14	11
2020-09	63			63	58
2020-10	385			385	488
2020-11	842			842	1251
2020-12	2076			2076	2163
2021-01	3696			3696	3399
2021-02	3819			3819	3170
2021-03	3468			3468	2806
2021-04	1745			1745	1256
2021-05	823			823	386
2021-06	327	3		330	71
2021-07	114	41		155	16
2021-08	31	56		87	9
2021-09	2	246		248	107
2021-10		869		869	450
2021-11		2818		2818	1462
2021-12		3137		3137	1558
2022-01		1494	102	1596	561
2022-02		481	1127	1608	532
2022-03		216	1875	2091	606
2022-04		49	1453	1502	292
2022-05			701	701	71
2022-06			275	275	31
2022-07			304	304	86
2022-08			571	571	127
2022-09			455	455	84
2022-10			664	664	144
2022-11			520	520	76
2022-12			445	445	91
Total	17495	9410	8492	35397	21395

Supplementary File 9: COVID-19 associated deaths and COVID-19 confirmed deaths for all age groups **(calculation of authors, Slovakia, 2024)**

Age	Deaths total	COVID-19 associated Deaths	COVID-19 confirmed Deaths
0	851	5	4
1	66	4	1
2	44	1	1
3	23	2	0
4	28	1	1
5	22	0	0
6	24	2	1
7	22	1	0
8	24	0	0
9	19	0	0
10	18	2	0
11	27	0	0
12	22	5	0
13	27	2	1
14	14	0	0
15	43	2	1
16	47	2	1
17	44	2	1
18	63	4	2
19	73	4	3
20	68	3	1
21	78	6	0
22	78	7	4
23	69	6	3
24	88	7	4
25	116	10	6
26	104	8	2
27	126	10	5
28	125	14	10
29	155	13	10
30	180	18	10
31	197	18	8
32	193	18	11
33	238	25	17
34	258	20	9
35	245	25	16
36	314	24	19
37	348	45	26
38	341	29	13
39	410	36	22
40	435	55	29

41	488	59	36
42	498	53	33
43	601	75	48
44	608	72	44
45	732	77	52
46	762	99	63
47	821	136	78
48	940	121	62
49	966	129	83
50	1056	147	91
51	1107	149	95
52	1132	148	97
53	1258	180	114
54	1380	202	129
55	1612	244	151
56	1805	289	187
57	1979	328	218
58	2089	313	194
59	2337	420	267
60	2474	446	278
61	2740	503	334
62	3039	570	374
63	3511	617	419
64	3746	666	441
65	3994	725	459
66	4213	807	500
67	4427	813	540
68	4737	916	614
69	4901	936	635
70	5210	1106	700
71	5160	1056	684
72	5121	1021	663
73	5285	1209	758
74	4976	1082	680
75	5039	1120	677
76	5023	1076	672
77	5239	1126	688
78	5410	1115	663
79	5671	1214	742
80	5682	1179	780
81	5742	1229	727
82	5722	1190	691
83	5712	1154	670
84	5609	1090	619
85	5607	1079	640

86	5429	1026	565
87	5214	971	500
88	5171	894	480
89	4708	796	440
90	4337	720	388
91	3609	595	271
92	3005	451	238
93	2430	339	177
94	1868	270	134
95	1438	202	100
96	1098	165	65
97	742	91	38
98	529	73	35
99	301	38	15
100	220	26	10
101	112	10	5
102	36	7	1
103	28	2	1
104	15	1	0
105	9	0	0
106	4	0	0
Total	192131	35399	21395
65+	148783	28920	17265
%	77,4%	81,7%	80,7%

Supplementary File 10: Age distributions for the three groups of COVID-19 confirmed deaths, COVID-19 associated deaths, and COVID-19 associated deaths that are not among the COVID-19 confirmed deaths (Men) **(calculation of authors, Slovakia, 2024)**

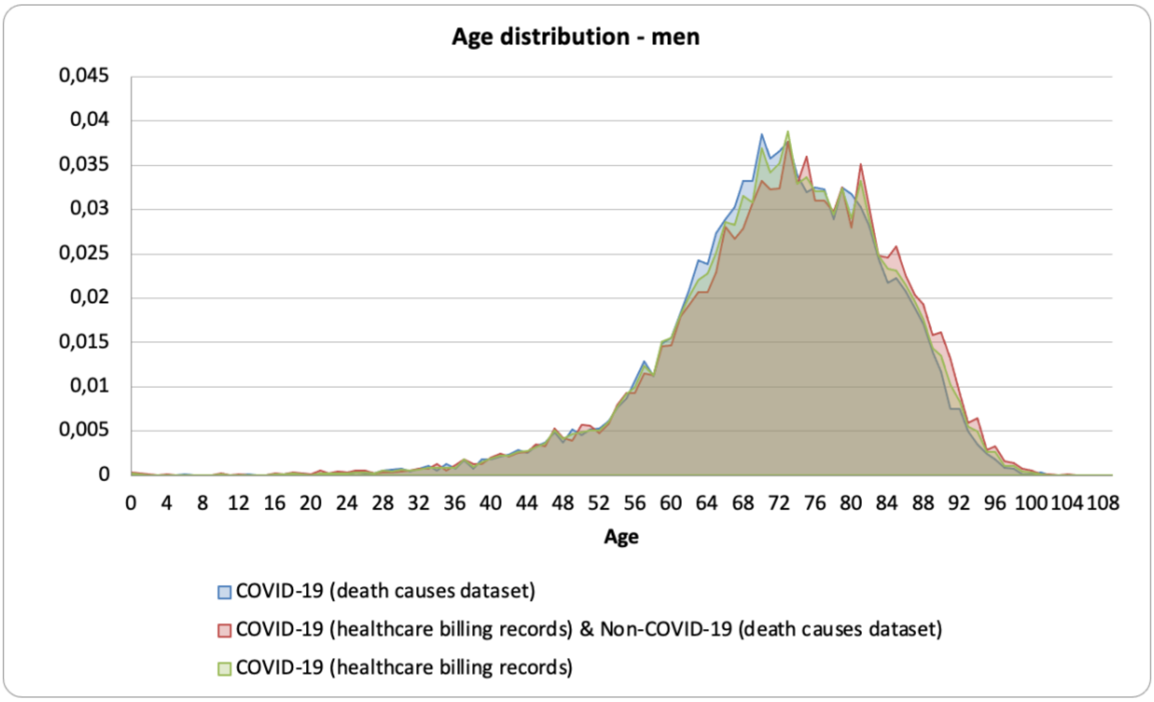

Supplementary File 11: Age distributions for the three groups of COVID-19 confirmed deaths, COVID-19 associated deaths, and COVID-19 associated deaths that are not among the COVID-19 confirmed deaths (Women) **(calculation of authors, Slovakia, 2024)**

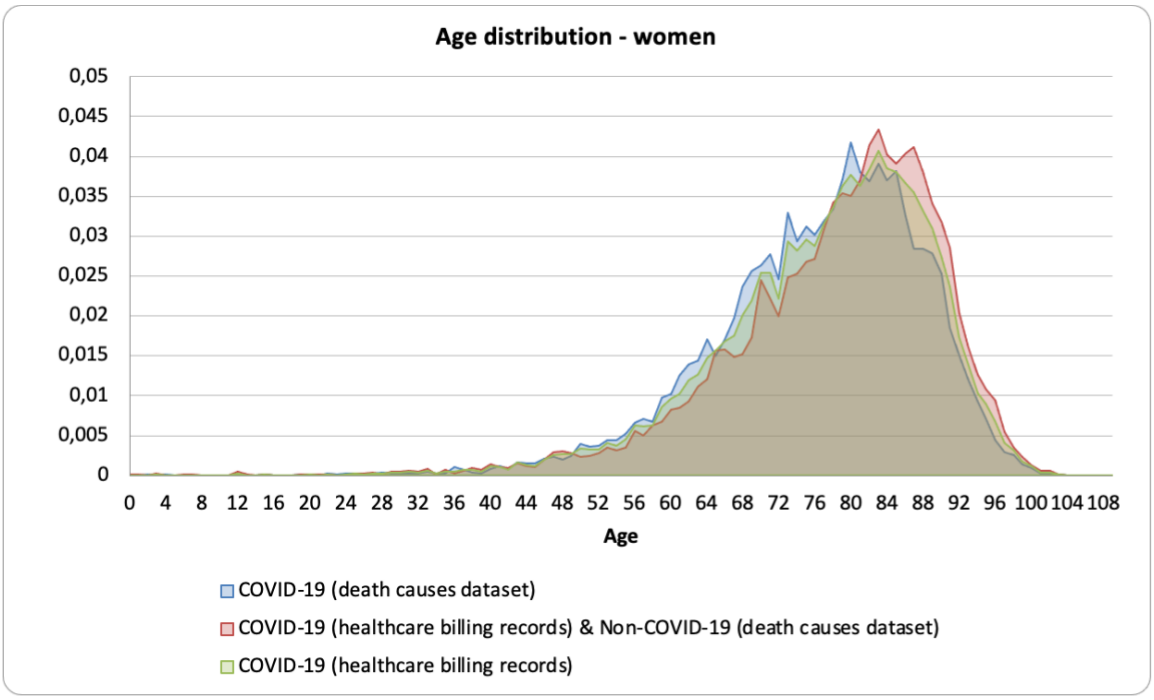

Supplementary File 12: Number of cases (Non-COVID-19 vs. COVID-19) according to methodological approach (healthcare billing records vs. death causes dataset) **(calculation of authors, Slovakia, 2024)**

**Year-Month**	**Non-COVID-19 (healthcare billing records) & Non-COVID-19 (death causes dataset)**	**COVID-19 (healthcare billing records) & Non-COVID-19 (death causes dataset)**	**Non-COVID-19 (healthcare billing records) & COVID-19 (death causes dataset)**	**COVID-19 (healthcare billing records) & COVID-19 (death causes dataset)**
2020-01	4991	0	0	0
2020-02	4690	0	0	0
2020-03	4989	5	1	0
2020-04	4242	15	7	18
2020-05	4199	25	0	5
2020-06	4041	15	1	0
2020-07	4269	6	0	1
2020-08	4417	8	4	7
2020-09	4247	22	17	41
2020-10	4793	108	211	277
2020-11	4549	251	659	592
2020-12	4603	600	687	1476
2021-01	4703	979	682	2717
2021-02	3746	1124	475	2695
2021-03	3768	1019	357	2449
2021-04	3635	621	132	1124
2021-05	3823	473	36	350
2021-06	4109	270	11	60
2021-07	4139	143	4	12
2021-08	4191	80	2	7
2021-09	4366	159	18	89
2021-10	4754	469	50	400
2021-11	4558	1497	141	1321
2021-12	4417	1726	147	1411
2022-01	4023	1078	43	518
2022-02	3505	1155	79	453
2022-03	3785	1563	78	528
2022-04	3310	1249	39	253
2022-05	3777	640	10	61
2022-06	4136	249	5	26
2022-07	4376	240	22	64
2022-08	4194	460	16	111
2022-09	3949	390	19	65
2022-10	4211	542	22	122
2022-11	4107	456	12	64
2022-12	5107	380	26	65
Total	152719	18017	4013	17382

Supplementary File 13: Structure of cases: Non-COVID-19 (healthcare billing records) & COVID-19 (death causes dataset) **(calculation of authors, Slovakia, 2024)**

Year-Month	Total	No U07.1,.3,.4 code in healthcare billing records	Beyond timeframe	Have U07.2 code (COVID-19 suspicion)
2020-03	1	1	0	0
2020-04	7	1	1	5
2020-06	1	1	0	0
2020-08	4	3	0	1
2020-09	17	6	0	11
2020-10	211	62	0	149
2020-11	659	277	0	382
2020-12	687	226	1	460
2021-01	682	241	8	433
2021-02	475	134	3	338
2021-03	357	96	5	256
2021-04	132	26	0	106
2021-05	36	10	4	22
2021-06	11	1	2	8
2021-07	4	1	1	2
2021-08	2	2	0	0
2021-09	18	5	0	13
2021-10	50	5	1	44
2021-11	141	36	5	100
2021-12	147	46	4	97
2022-01	43	9	1	33
2022-02	79	23	3	53
2022-03	78	18	6	54
2022-04	39	6	1	32
2022-05	10	1	5	4
2022-06	5	0	1	4
2022-07	22	6	1	15
2022-08	16	4	5	7
2022-09	19	3	0	16
2022-10	22	4	3	15
2022-11	12	2	3	7
2022-12	26	4	5	17
Total	4013	1260	69	2684

Supplementary File 14: Excess COVID-19 deaths in Slovakia and the United States **(calculation of authors, Slovakia, 2024)**

	**USA March-May 2020 (34)**	**USA all of 2020 (33)**	**Slovakia 2020-2022**	**Slovakia 2020**	**Slovakia 2021**	**Slovakia 2022**
Total number of deaths	781,000	2,801,439	192,133	59,089	73,461	59,583
Excess deaths	122,300	522,368	31,789	5,641	20,013	6,135
COVID-reported deaths	95,235	378,039	21,496	4,004	14,769	2,723
Difference between Excess deaths and COVID-reported deaths (possible absolute underestimation)	27,065	144,329	10,293	1,637	5,244	3,412
Difference between Excess deaths and COVID-reported deaths (possible relative underestimation)	28.4%	38.2%	47.9%	40.9%	35.5%	125.3%
